# Enhancing patient navigation to improve intervention session attendance and viral load suppression of persons with HIV and substance use: a secondary post hoc analysis of the Project HOPE study

**DOI:** 10.1186/s13722-017-0081-1

**Published:** 2017-06-27

**Authors:** Maxine Stitzer, Tim Matheson, Colin Cunningham, James L. Sorensen, Daniel J. Feaster, Lauren Gooden, Alexis S. Hammond, Heather Fitzsimons, Lisa R. Metsch

**Affiliations:** 1 Department of Psychiatry and Behavioral Sciences, Hopkins Bayview Medical Center, Johns Hopkins University School of Medicine, 5510 Nathan Shock Drive, Baltimore, MD 21224 USA; 2 San Francisco Department of Public Health, 25 Van Ness Avenue Suite 500, San Francisco, CA 94102 USA; 3 UCSF Department of Psychiatry, Zuckerberg San Francisco General Hospital and Trauma Center, 1001 Potrero Avenue SFGH Building 20, Rm. 2117, San Francisco, CA 94110 USA; 4 Department of Public Health Sciences, University of Miami Miller School of Medicine, 1120 Northwest 14th Street, CRB 1059, Miami, FL 33136 USA; 5 Department of Sociomedical Sciences, Mailman School of Public Health, Columbia University, 722 West 168th Street, Room 918, New York, NY 10032 USA

**Keywords:** HIV health care, HIV substance users, Patient navigation, Contingent incentives, Session attendance, Vial suppression

## Abstract

**Background:**

Interventions are needed to improve viral suppression rates among persons with HIV and substance use. A 3-arm randomized multi-site study (Metsch et al. in JAMA 316:156–70, 2016) was conducted to evaluate the effect on HIV outcomes of usual care referral to HIV and substance use services (N = 253) versus patient navigation delivered alone (PN: N = 266) or together with contingency management (PN + CM; N = 271) that provided financial incentives targeting potential behavioral mediators of viral load suppression.

**Aims:**

This secondary analysis evaluates the effects of financial incentives on attendance at PN sessions and the relationship between session attendance and viral load suppression at end of the intervention.

**Methods:**

Frequency of sessions attended was analyzed over time and by distribution of individual session attendance frequency (PN vs PN + CM). Percent virally suppressed (≤200 copies/mL) at 6 months was compared for low, medium and high rate attenders. In PN + CM a total of $220 could be earned for attendance at 11 PN sessions over the 6-month intervention with payments ranging from $10 to $30 under an escalating schedule.

**Results:**

The majority (74%) of PN-only participants attended 6 or more sessions but only 28% attended 10 or more and 16% attended all eleven sessions. In contrast, 90% of PN + CM attended 6 or more visits, 69% attended 10 or more and 57% attended all eleven sessions (attendance distribution χ^2^[11] = 105.81; p < .0001). Overall (PN and PN + CM participants combined) percent with viral load suppression at 6-months was 15, 38 and 54% among those who attended 0–5, 6–9 and 10–11 visits, respectively (χ^2^(2) = 39.07, p < .001).

**Conclusion:**

In this secondary post hoc analysis, contact with patient navigators was increased by attendance incentives. Higher rates of attendance at patient navigation sessions was associated with viral suppression at the 6-month follow-up assessment. Study results support use of attendance incentives to improve rates of contact between service providers and patients, particularly patients who are difficult to engage in care.

*Trial Registration* clinicaltrials.govIdentifier: NCT01612169.

## Background

Inconsistent engagement with health care services is common among individuals with substance use disorders [2]. This is especially problematic for people living with HIV, due to the importance of ongoing engagement in care for optimal health outcomes. Because drug use can interfere with every step of the treatment cascade [3], there is evidence that HIV-infected drug users may have faster disease progression, higher risk of acquiring new AIDS-defining conditions [4] and higher rates of hospitalization [5] compared to non-drug users. Azar et al. [6] came to similar conclusions in a review of studies that addressed the associations among Alcohol Use Disorder (AUD), health care utilization, adherence to antiretroviral medications, and HIV treatment outcomes. These findings underscore the importance of focused engagement in care interventions for vulnerable populations with substance use.

Contingency management (CM) in the form of financial incentives for uptake and adherence to health care services has promising results in a variety of settings [7]. Examples of simple but effective attendance interventions include financial incentives for return to a test site to receive HIV [8] or tuberculosis [9, 10]) test results, completion of a 3-injection course of hepatitis B vaccine [11] return to a substance use disorders treatment program following intake to complete an individualized service plan [12] and persistence with substance use disorders treatment when modest financial incentives were offered for a return visit to the clinic following intake and for attendance on day 5 post-admission [13].

Studies targeting sustained attendance at therapy visits have also shown contingent financial incentives to be efficacious in improving engagement in HIV care among persons with substance use disorders [14, 15]. Other studies using a variety of specific incentive delivery methods report that incentives improve attendance at counseling sessions [16–22] or psychiatric services [23] in substance use disorders treatment programs. However, studies to date have not shown the effectiveness of contingency management in improving health-related outcomes including viral load suppression among HIV-positive substance users [1, 7, 15].

A recently completed 3-arm multi-site study conducted within the National Drug Abuse Treatment Clinical Trials Network (CTN 0049/Project HOPE: Hospital as Opportunity for Prevention and Engagement for HIV Infected Drug Users) provided an opportunity to examine the potential value of adding incentives to a behavioral intervention platform in improving HIV outcomes among substance users with uncontrolled HIV. CM was incorporated into a patient navigation (PN) intervention designed to improve engagement in HIV care and substance use treatment and adherence with HIV health care regimens among persons with HIV and substance use. Patient navigation is a clinical support intervention that uses motivational interviewing techniques and a flexible, problem-solving approach to overcoming barriers [24, 25] with the aim of promoting engagement in health care services. A navigation approach has been previously shown effective for improving linkage to [26] and retention in [27] HIV care. Outcomes for the navigation intervention with (PN + CM) and without (PN only) incentives was compared to a usual care referral group.

The primary outcome paper from the HOPE study [1] showed no difference among the 3 study arms at the primary 12-month endpoint, 6-months after the intervention ended. However, a secondary analysis showed that at 6 months, immediately after conclusion of the interventions, rates of viral load suppression were 38.2, 43.1 and 50.4% in usual care, PN-only and PN + CM, respectively with PN + CM rates being significantly (p = .03) higher compared to the usual care control. The present secondary post hoc analysis expands on these findings by analyzing attendance incentive effects over time during the intervention, differences in session attendance for PN versus PN + CM, and the relationship between PN contact and viral load suppression at the 6-month outcome time point.

## Methods

The HOPE study enrolled and randomized 801 persons with HIV and substance use recruited from 11 hospitals across the US. More detail on study methods as well as participant characteristics can be found in the primary outcome manuscript [1]. The study was approved by local IRBs at each participating institution. Human subjects signed informed consent prior to participation. Eligibility criteria included having a detectable HIV viral load and evidence of (in medical records) any opioid, stimulant (cocaine, amphetamines, ecstasy) or heavy alcohol use within the past year. Participants were randomly assigned in a 3-arm design to receive standard of care which typically included referral to HIV and substance use services or one of two patient navigation interventions delivered with (PN + CM; N = 271) or without (PN only; N = 266) a multi-target incentive program. Participants in both PN conditions receive the same PN intervention lasting 6 months with 11 sessions specified in the protocol. During the sessions, navigators used motivational interviewing techniques to assist participants to draw on their own capabilities and resources while specifically encouraging them to engage in HIV care, initiate or reinstate antiretroviral therapy and take steps to reduce or stop their substance use, potentially including entry into substance use disorders treatment. Session schedules were flexible in both timing and location, with the intent that they be more frequent during early months of the intervention and less frequent in later months.

PN + CM participants could earn up to a total of $1160 during the 6-month intervention by meeting target goals on 8 different behaviors related to HIV treatment engagement and substance use abatement. Behavior targets included attending HIV care doctor visits, providing evidence of an active anti-retroviral medication prescription, entering substance abuse treatment and providing drug negative urine samples at PN visits. Details of the multi-target CM plan and rationale have been described [28]. For attendance at the 11 PN sessions, one of the components of the full multi-target incentive plan, a total of $220 (19% of total possible earnings) could be earned under an escalating schedule that increased by $2 for each successive session attended from $10 for session 1 to $30 for session 11. Payment was made for all sessions independent of when or where they occurred. Participants could receive earnings immediately or hold them in an account for receipt at a later time. Payment was made in cash (4 sites) or debit card transfer (1 site), in gift cards to local retail establishments (4 sites) or with a combination of cash and gift cards (2 sites; one using patient choice).

For this analysis, the PN + CM and CM conditions were compared on sessions attended over the 6-month intervention period and on HIV viral load suppression, at 6 months. The 6-month outcome time point was selected for this post hoc analysis because this outcome assessment occurred directly after completion of the intervention period. The Wilcoxon test for difference in medians is used to test between group (PN-only vs PN + CM) differences in attendance frequency due to the non-normal distribution of attendance frequency. We report the normal-approximation to the Wilcoxon test statistic due to the relatively large sample [29]. Categorical comparisons are tested using Chi square. Attendance frequency categories (0–5, 6–9, 10–11 sessions attended) used to analyze the association with viral suppression outcomes are based on empirical examination of obtained attendance frequency distributions.

## Results

**Fig. 1:**
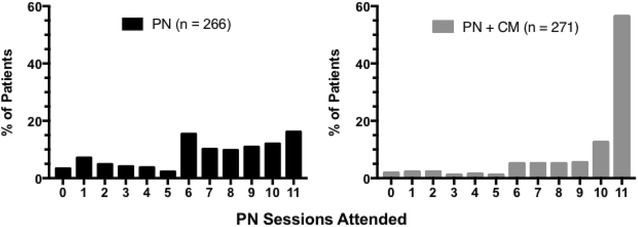
The contrasting distribution of PN visit attendance for participants in the PN (N = 266) and PN + CM (N = 271) treatment groups. *Bars* indicate the percentage of participants in the designated treatment group who achieved each total number of PN visits from 0 to 11 during a 6-month intervention. Incentives were available on an escalating scale starting at $10 and increasing to $30 per visit; PN + CM could earn a total of $220 for attending all visits

Figure 1 shows the significantly different distribution of session attendance frequency for PN only and PN + CM participants (χ^2^ [11] = 105.81; p < .0001). There is a sharp peak of attendance in the PN + CM group where 56.5% of participants attended all eleven sessions, 69% attended 10 or more and 90% attended 6 or more sessions. The contrasting distribution in the PN only group shows that the majority (74.4%) attended 6 or more sessions but only 28.2% attended 10 or more and 16.2% attended all eleven sessions. Median number of sessions attended differed significantly between the two groups (z = −9.8, P < .0001). Median sessions attended was 7 (interquartile range [IQR], 5–10) for the PN-only group versus 11 (IQR, 8–11) in the PN + CM group.

**Fig. 2:**
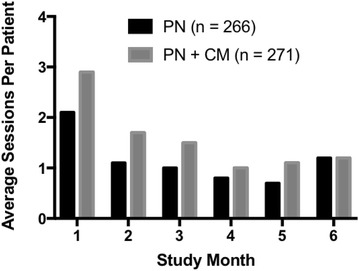
shows mean number of PN visits attended per month during the 6-month intervention for PN (N = 266) and PN + CM (N = 271) participants

Figure 2 shows how sessions were distributed over time (mean sessions per month) for the two groups. Between group differential was greatest in the first month when PN + CM participants attended nearly one whole visit more than navigation only participants (2.9 vs 2.1 visits). Frequency of visits declined over months 1–5 in both groups, but mean visits remained higher for the PN + CM than for PN only group throughout this time period (χ^2^[5] = 24.03; p −.0002).

**Fig. 3:**
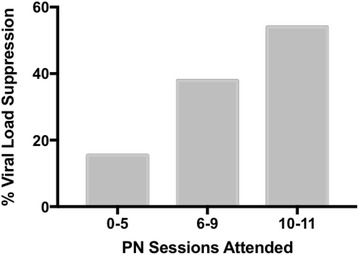
The percent of all participants collapsed across PN and PN + CM (N = 508 due to missing viral load data) with suppressed viral load (≤200 copies/mL) at the 6-month assessment as a function of PN visits attended. Number of visits attended has been divided into 3 functional categories: low (0–5 visits; N = 78), moderate (6–9 visit; N = 169) and high (10–11 visits; N = 261)

As shown in Fig. 3 with data combined for the two groups, there was a linear relationship between PN visit attendance and viral load suppression. Among those attending 0-5 sessions, only 15.4% were virally suppressed at 6 months. Suppression rate more than doubled, rising to 37.9% among those attending an intermediate number of sessions (6–9). The highest rate of viral load suppression at 54% was seen in those who attended at least 10 of the 11 possible sessions (total sample χ^2^(2) = 39.07, p < .001). Table 1 shows that this relationship was apparent for the PN (χ^2^(2) = 14.72, p < .001) and PN + CM (χ^2^(2) = 24.35, p < .001) groups separately as well.

**Table 1:** Viral load outcomes at end of treatment (month 6) by number of PN visits attended

Visits attended	PN		PN + CM	
	N	% with viral suppression	N	% with viral suppression
0–5	57	19.3	21	4.8
6–9	116	40.5	53	32.1
10–11	75	52.0	186	54.8
Total	248		260	

## Discussion

As previously reported [1], attendance incentives embedded in a multi-target contingency management program for persons with HIV and substance use increased contact between participants and their assigned navigators. Our analysis expanded on median differences previously reported. The most notable finding is in the number of patients attending all eleven of the possible scheduled sessions. Rates of full attendance were 3.5 times higher for PN + CM than for PN only participants. The increase in visit frequency for navigation only in month 6 is likely related to the opportunity to complete 6-month data collection for additional payment at that time.

The results are consistent with previous literature demonstrating that contingent financial incentives are effective for improving contact with services. Here, we also demonstrate a significant association between viral suppression and rates of attendance independent of whether incentives were used in the PN protocol. Specifically, in the combined groups, the more sessions attended, the more likely participants were to have viral suppression. This relationship suggests that the PN intervention was a useful part of the overall strategy for achieving the desired health outcome, with role of the incentives being to increase contact with the PN services. If the PN intervention accomplished it’s aims, we would expect to see higher rates of engagement in HIV care and substance abuse treatment among PN + CM compared to PN only. This prediction will be examined in subsequent secondary analyses. The potential mediating influence of PN contact on viral load outcomes can also be further elucidated in multivariable analysis that takes into account other potential mediating and moderating variables including levels of on-going substance use. However, since multiple behaviors were incentivized in this protocol, it will not be possible to disentangle the independent mediating variable of PN contact, which could be done if PN contact were the only behavior incentivized.

While the relationship between session attendance and HIV viral suppression was strong, it is also notable that nearly half the participants who attended 10 or 11 PN sessions were not virally suppressed at 6 months despite their high rate of contact with the PN intervention. This suggests that further examination of the project HOPE data is needed to identify areas where either the PN or CM intervention or both could be further improved. This could involve prolonging or intensifying the intervention, increasing incentive amounts or altering their distribution across target behaviors. New intervention features may also be useful such as in-hospital initiation of medication treatments for HIV and/or substance abuse.

Several features of the study may have affected outcomes. These include features of the appointment scheduling specified in the study protocol, the number and type of appointment reminders made by PNs to their participants, and details of the incentive program. For example, the difference between rates of attendance for PN versus PN + CM may have been even greater in a protocol where PN session number was not constrained. It is likely that providing the incentives immediately at PN sessions contributed to the effectiveness of the present intervention in promoting attendance at PN sessions. Further it is possible that the escalating schedule of reinforcement that provided higher incentives for attendance at later sessions played a role in supporting the full attendance observed in over half of the PN + CM participants., However, only a single set of attendance incentive parameters was tested (i.e. incentive amounts and method of delivery) and it is possible that rates of attendance could have been further improved with higher valued incentives, or that equivalent or better results could be obtained with use of other incentive delivery methods such as the prize draw method [30] or with fixed rather than escalating incentive values for successive attendance. More research on these parameters would be desirable.

This study showed that attendance incentives substantially improved rates of contact between persons with HIV and substance use and their patient navigators who delivered a strength-based intervention designed to encourage re-engagement into HIV health care and substance use services. The association between attendance and viral load suppression outcome is encouraging as it suggests that contact with the PN intervention was effective for improving this important health outcome. Study results support use of attendance incentives within the health care system to improve rates of contact between service providers delivering beneficial interventions and patients who need services, particularly patients who are difficult to engage in care due to untreated substance use.

## Abbreviations

CM: contingency management
PN: patient navigation
